# Hepatic artery lymph node relevance in periampullary tumors: A retrospective analysis of survival outcomes

**DOI:** 10.3389/fsurg.2022.963855

**Published:** 2022-12-06

**Authors:** Danny Conde, Carlos Rey, Manuel Pardo, Andrea Recaman, Juan Carlos Sabogal Olarte

**Affiliations:** ^1^ Department of Hepatobiliary and Pancreatic Surgery, Hospital Universitario Mayor Méderi, Bogotá, Colombia; ^2^ School of Medicine, Universidad el Rosario, Bogotá, Colombia; ^3^ Chief and Chairman of Hepatobiliary and Pancreatic Surgery Department, Hospital Universitario Mayor Méderi, Bogotá, Colombia

**Keywords:** periampullary tumors, hepatic artery lymph node, prognostic factor, disease-Free survival, pancreaticoduodenectomy, pancreatic adenocarcinoma.

## Abstract

**Background:**

The Periampullary area comprehends a heterogeneous and complex structure with different histological tissues. Surgical standards include the peripancreatic regional lymphadenectomy, and during pancreatoduodenectomy (PD) the hepatic artery lymph node HALN(8a) is dissected. We aimed to describe the prognostic significance of the HALN(8a) lymph node metastasis in terms of disease-free survival (DFS) and overall survival (OS) in a specific cohort of patients in limited economic and social conditions.

**Methods:**

A retrospective study was conducted based on a prospective database from the HPB department of patients who underwent pancreaticoduodenectomy (PD) due to periampullary tumors during 2014–2021. Overall survival (OS) and disease-free survival (DFS) were estimated to be associated with positive HALN(8a) using Kaplan-Meier analysis. Log Rank test and Cox proportional hazards regression analysis was used.

**Results:**

111 patients were included, 55,4% female. The most frequent pathology was ductal adenocarcinoma (60.3%). The positive rate of the HALN(8a) node was 21.62%. The Median OS time was 25.5 months, and the median DFS time was 13,8 months. Positive HLAN(8a) node, the cutoff of lymph node ratio resection (LNRR), and vascular invasion showed a strong association with OS. (CoxRegression *p* = 0.03 HR 0.5, *p* 0.003 HR = 1.8, *p* = 0.02 HR 0.4 CI 95%). In terms of DFS, lymph node ratio cutoff, tumoral size, and vascular invasion showed a statistically significant association with the outcome (*p* = 0.008, HR = 1.5; *p* = 0.04 HR = 2.1; *p* = 0.02 HR = 0.4 CI 95%).

**Conclusion:**

In this series of PD, OS was reduced in patients with HALN(8a) compromise in patients with pancreatic cancer, however without statistical significance in DFS. In multivariate analysis, lymph node status remains an independent predictor of OS and DFS. Further studies are needed.

## Introduction

The periampullary region is a complex area, composed of different histologic tissues, namely, biliary, duodenal, pancreatic, and ampullary. It is a frequent site of tumor growth; they correspond approximately to 5% of all gastrointestinal malignancies. Among the different types, pancreatic adenocarcinoma is the most frequent (70%), followed by ampullary cancers (15%–25%). Duodenal and distal common bile duct tumors (CBD) are present in approximately 10% (1). In rare cases, metastases from another primary cancer may be located in the periampullary region. The most frequent cancer type is renal cell carcinoma (3). Periampullary cancers arising from the duodenum, ampulla, and CBD are associated with better survival than pancreatic cancer and this may be related to tumor biology and more aggressive behavior (2).

Differentiation among histologic types preoperatively is difficult due to the proximity of the structures and their similar immunophenotype. Molecular characterization can be used for objective classification and targeted therapy (3, 4–6) However, despite their anatomical proximity and similar operative approach, these cancers have demonstrated a large disparity in outcomes. Currently, the most studied prognostic factors associated with disease-free survival (DFS) and overall survival (OS) are the resection margins, tumoral biology, vascular invasion, and lymph node metastasis (4–6).

The gold standard of treatment is pancreatoduodenectomy in all suspected or confirmed malignancy cases. During this procedure, resection of the hepatic artery node is performed routinely in most places to dissect and visualize the emergency of the gastroduodenal artery but is not mandatory in lymph node dissection (4, 8–10). The 8th edition of the TNM classification recommends pathologic examination of at least 12 nodes for reliable assessment of the lymphatic status (3). It has been identified in previous pancreatic cancer studies that the compromise of the hepatic artery lymph node (HALN), also called station 8a, a second-echelon node according to the Japan Pancreas Society classification (7) can be related to a reduction of OS (11). In fact, some case series report that the prognosis of HALN(8a) metastases can be compared with peritoneal carcinomatosis or liver metastases (10).

However, to our knowledge, studies evaluating the relevance of this node in other periampullary tumors are scarce. Additionally, no research studies have been published on patients from central and South America. The public health system in Colombia, like in the majority of countries in south and central America, is mixed with a significant private component with intermediation. This fact causes an important limitation to access to the health system and in this particular scenario, it is one of the biggest challenges to face with patients suffering from periampullary cancer who are not able to access adequate care such as neoadjuvant therapy at the right time. This is an interesting opportunity not only to understand but also to show some accurate data that will help to develop our own understanding of the specific social and economic conditions that are far from those in North America and Europe.

Given the different perspectives on the relation of HALN(8a) positive and poor prognosis, this retrospective study is proposed to assess the oncologic impact of HALN(8a) in our series of periampullary tumors taken to pancreaticoduodenectomy in Bogotá—Colombia.

## Materials and methods

### Patients

**Figure 1:**
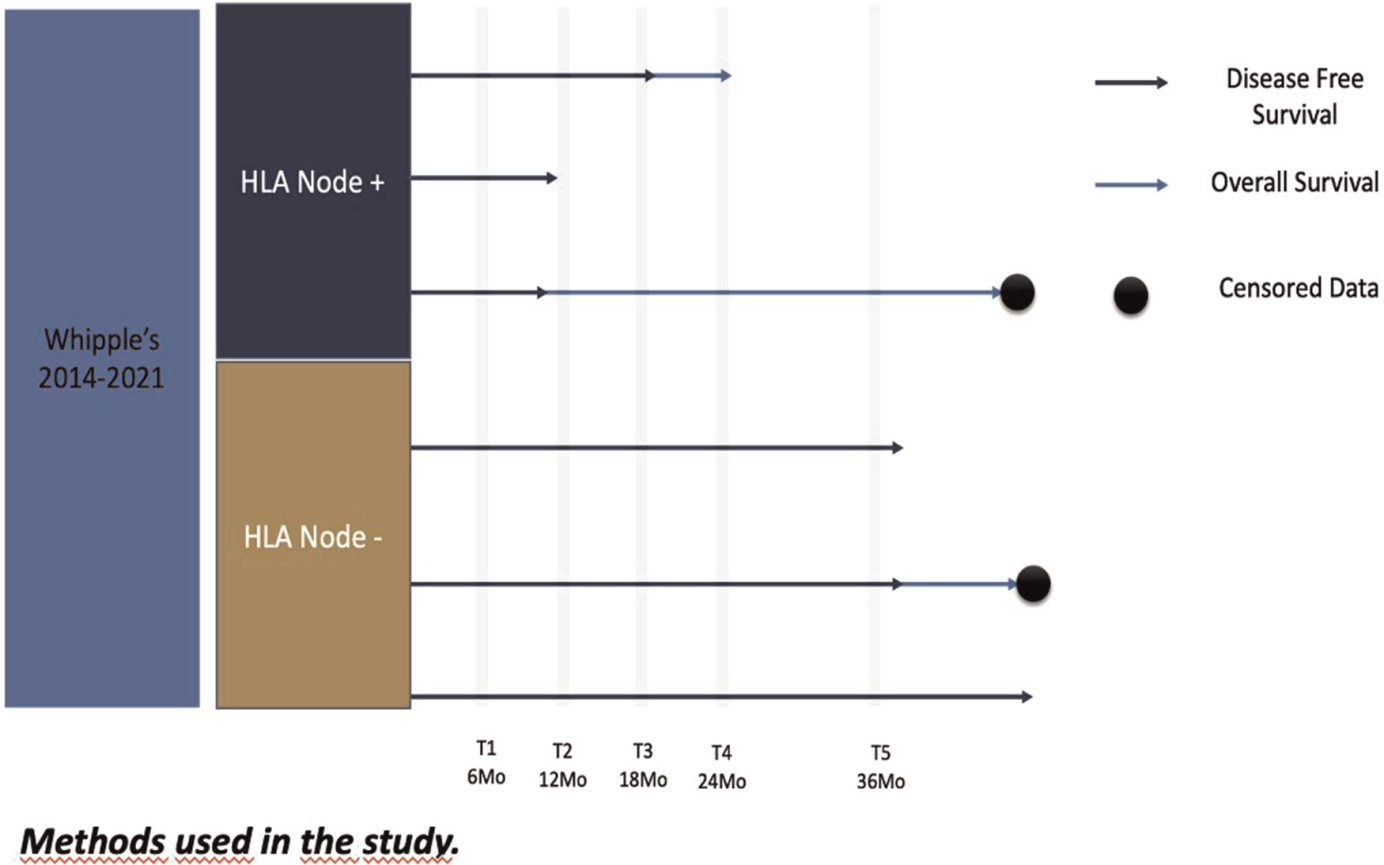
Methodology.

After the institutional review board and Ethical Committee approval, a retrospective cross-sectional review of patients of the single institution of the hepatic-pancreato-biliary database was performed. Patients who underwent pancreatoduodenectomy for periampullary tumors during 2014–2021 were included in this study (Figure 1). All these cases were treated with upfront surgery given its resectability or in some selected cases (by a multidisciplinary board) of borderline tumors according to the social and health care conditions. None of these borderline patients received neoadjuvant therapy because of limited healthcare accessibility or board institutional decisions. Pathologic reports were reviewed to identify hepatic node resection, oncological margins, and lymph nodes harvested.

Cases with incomplete data, positive macroscopic margins (R2), and not-reported hepatic nodes in pathologic specimens were excluded from the analysis. A descriptive analysis was made by calculating the proportions and frequencies of qualitative variables and central tendency measures for quantitative variables.

Variables were analyzed according to factors that have been shown relevance in previous studies for periampullary tumors: tumor size, histologic grade, vascular invasion, and completeness of resection.

### Statistical analysis

#### Overall survival and disease-free survival

OS was defined from the date of the surgery until June 2021. Disease-free survival was defined as the time from the postoperative period, to the first documentation of tumoral relapse such as clinical (ascites), biomarkers analysis (Ca 19-9 antigen elevation), or radiographic findings (tomographic evidence of local or distance relapse) until June 2021. Survival analysis was performed using the Kaplan-Meier method for OS and DFS. LogRank tests were used to examine the association of clinical and pathological variables with the OS and DFS. Differential analysis was performed: patients that receive adjuvant therapy vs. HALN(8a) node compromise or not. Significant characteristics (*p*-value < 0.20) in LogRank tests were included in a multivariate cox proportional hazard regression model. Data with a minor or equal *p*-value of 0.05 were considered statistically significant. Statistical analysis for the comparison of mean values will be performed using *t*-tests or Wilcoxon-Mann-Whitney tests as appropriate. Assumptions regarding normal distribution will be checked by graphic representation, Shapiro-Wilk-Tests, and Q-Plots, respectively. The Chi-squared and Fisher's exact tests were used to compare categorical variables, as appropriate. Statistical analysis will be carried out using the Statistical Package of STATA Version 17.0 BE-Basic Edition (StataCorp LLC StataCorp 4,905 Lakeway Drive College Station, Texas 77,845 USA). A two-tailed *p*-value of < 0.05 will be considered statistically significant.

#### Lymph node ratio resection (LNRR)

A lymph node ratio resection (LNRR) was established as the main variable. LNRR is defined as the ratio between the total lymph node resected and the positive ones in the pathology report. A cutoff value was generated for this variable with logarithmic regression after Kaplan-Meier estimates, and a ROC value of 0.6 was estimated for the LNRR cutoff for the study population.

#### Follow-up

By institutional protocol, follow-up was performed at 6, 12, 18, 24, 30, and 36 months to determine the time of tumoral relapse, using tumoral biomarkers, clinical assessment, and tomographic studies. Mortality was determined by the time of deaths reported in the national database.

#### Lymph node subgroup analysis

Lymph node retrieval was classified according to peripancreatic and HALN positivity: Peripancreatic (+)/HALN (+) = 18.92% (*n* = 21/111), Peripancreatic (+), HALN (−) = 29.73% (*n* = 33/111), and Peripancreatic (−), HALN (−) = 48.65% (*n* = 54/111).

## Results

### Patients and tumor characteristics

A total of 136 patients underwent PD resection for pancreatic cancer during 2014–2020. Twenty-five patients were excluded from the study because of incomplete data. A total of 111 patients were included. The overall positive rate of lymph node resection, HALN(8a) positive rate, and the LNRR were analyzed for each patient.

Females constituted 54,05% (*n* = 60) of cases. The mean age at the time of resection was 63.11y (SD 10.7). Pre-operative biomarker CA—19 9 was measured with a mean of 480 ng/dl (IQR 125;480). Other patient characteristics are summarized in Table 1. All patients underwent standard PD without pylorus preservation. The pathology in most of the patients was ductal adenocarcinoma at 60.36% (*n* = 67) followed by 22.52% for tumors of the ampulla of Vater. A moderate grade of differentiation of the tumor was found in 57% (*n* = 64) of the patients and a well-differentiated pathology (31.5%, *n* = 35).

**Table 1:** Patient demographics.

Variable	Value
**Demographic Characteristics**		
Sex. *N* (%)	Male	51 (45,95)
	Female	60 (54,05)
Age (Mean)	Male	63,2 year
	Female	56,5 year
Preoperative CA 19 9 mean (IQR)		480,8 (IQR 125;480)
Body mass index median (SD)		23,6 (4,29)
Diabetes mellitus *N* (%)		19 (17,12)
Smoker *N* (%)		13 (11,71)

Patients were classified using NCCN criteria. From the population of the study, 94.6% (*n* = 105) presented a resectable disease, the remaining patients were classified as borderline tumors. No locally advanced patients were included. R0 resections were achieved in 95,05% of the patients (*n* = 106); The majority of the patients have a vascular and perineural invasion in the pathology report (60,36% and 68,47% respectively). (See Table 2). The mean number of harvested lymph nodes was 14.53 (SD 5.8). The overall positive rate of the HALN(8a) node was 21.62% (*n* = 24). The overall positive nodes harvested was 9,01% (179 nodes out of 1613). The LNRR mean was 0.45 (SD 0.05). In the postoperative period, 42.34% (*n* = 47) do not receive adjuvant therapy due to both health insurance issues and personal decisions. During the analysis, the maximum time that patients receive chemotherapy after surgery was 24 months. 62,5% (*n* = 15) of the patients with positive HALN(8a) receive postoperative chemotherapy. Tumoral relapse was documented in 53.15% of the patients at 5 years of follow-up.

**Table 2:** Pathologic characteristics.

Variable	Value
**Tumor Characteristics**		
Tumoral size *N* (%)	<2 cm	19 (17,12)
	>2 cm	92 (82,88)
Resectability criteria *N* (%)	Resectable disease	94,6 (105)
	Borderline disease	5,4 (6)
Type of tumor *N* (%)	Ductal adenocarcinoma	67 (60,36)
	Ampullary tumors	25 (22,5)
	Cystadenocarcinoma	5 (4,50)
	Other	14 (12,64)
Differentiation grade *N* (%)	Well differentiated	35 (31,53)
	Moderate grade	64 (57,66)
	Poorly differentiated	10 (9,01)
	Undifferentiated	2 (1,80)
Adjuvant therapy *N* (%)	Received	64 (57,66)
Pathology report		
	Vascular invasion *N* (%)	67 (60,36)
	Perineural invasion *N* (%)	76 (68,47)
	R0 margins *N* (%)	106 (95,05)
	Positive rate of HLA node *N* (%)	24 (21,62)
Tumoral relapse at 5 years %		55,8

### Survival analysis

The median follow-up for all the patients was 19.98 months. The median OS time was 25,5 months (IQR 12,5–84), and the median DFS time was 13.58 months (IQR 7,5–57). For patients that received adjuvant chemotherapy (57,66% *n* = 64/111), the median OS time was 16,3 months (IQR 5,5–84,6) and the median DFS time was 16 months (IQR 7,5–57), with a maximum of 57 months. The relationship between preoperative CA 19-9 value and hepatic artery lymph node positivity was evaluated, and a mean comparison was performed, there is a slight difference between the groups (HALN(8a)—515,1; HALN(8a) +  = 354,3); two-way *t*-test was performed, however, there is any statistical relationship between CA 19-9 and HALN(8a) positivity (*p*-value = 0.42 CI 95%). The 5-year mortality and disease recurrence were analyzed in the most frequent pathology reports; ductal adenocarcinoma was related to an increased risk of disease recurrence and overall survival with statistical significance compared with ampullary tumors. (DFS: *p* = 0.008 CI 95% 1.21–3.70 HR 2.1; OS: *p* = 0.000 CI 95% 0.2–1.2 HR 1.5). The median overall survival in patients with ductal adenocarcinoma was 6.39 (IQR 6–7.1), and median disease-free survival in this group of patients was 9.97 months (IQR 7.6–11.2). For the ampulla of Vater tumors, the median OS was 7.7 (IQR 5.6–9.5); in terms of DFS, the median was 19,75 (IQR 12,5–24.4).

As well, pathology groups were classified according to the HALN(8a) positivity. In 26.8% (*n* = 18) of cases with ductal adenocarcinoma, hepatic artery lymph nodes were evidenced positive; in contrast patients with ampulla of Vater carcinoma, the HALN(8a) rate of positivity was 8% (*n* = 2). Median overall survival in patients with ductal adenocarcinoma with HALN(8a) + was slightly minor compared with HALN(8a)—group (Median 5.96 (IQR 4.5–7.8) vs. Median 6.5 (IQR 5.2–7.9) respectively). In terms of DFS, there is a difference as well comparing these two groups, in patients with HALN(8a) + median was 9.08 (IQR 7.4–11.2) and for patients with negative HALN(8a) median DFS was 10.34 (IQR 9.1–11.9).

In the group of the ampulla of Vater carcinoma with positive HALN(8a), the overall survival median was 5.56 (IQR 3.2–8.9) compared with the negative node group with a median of 7.96 (IQR 5.2–11.23). For disease-free survival in cases with positive HALN(8a) median was 4.5 (IQR 3.5–8.3), in the negative group, there is a greater difference with a median of 22.8 (IQR 17.2–27.5).

### Overall survival

**Figure 2:**
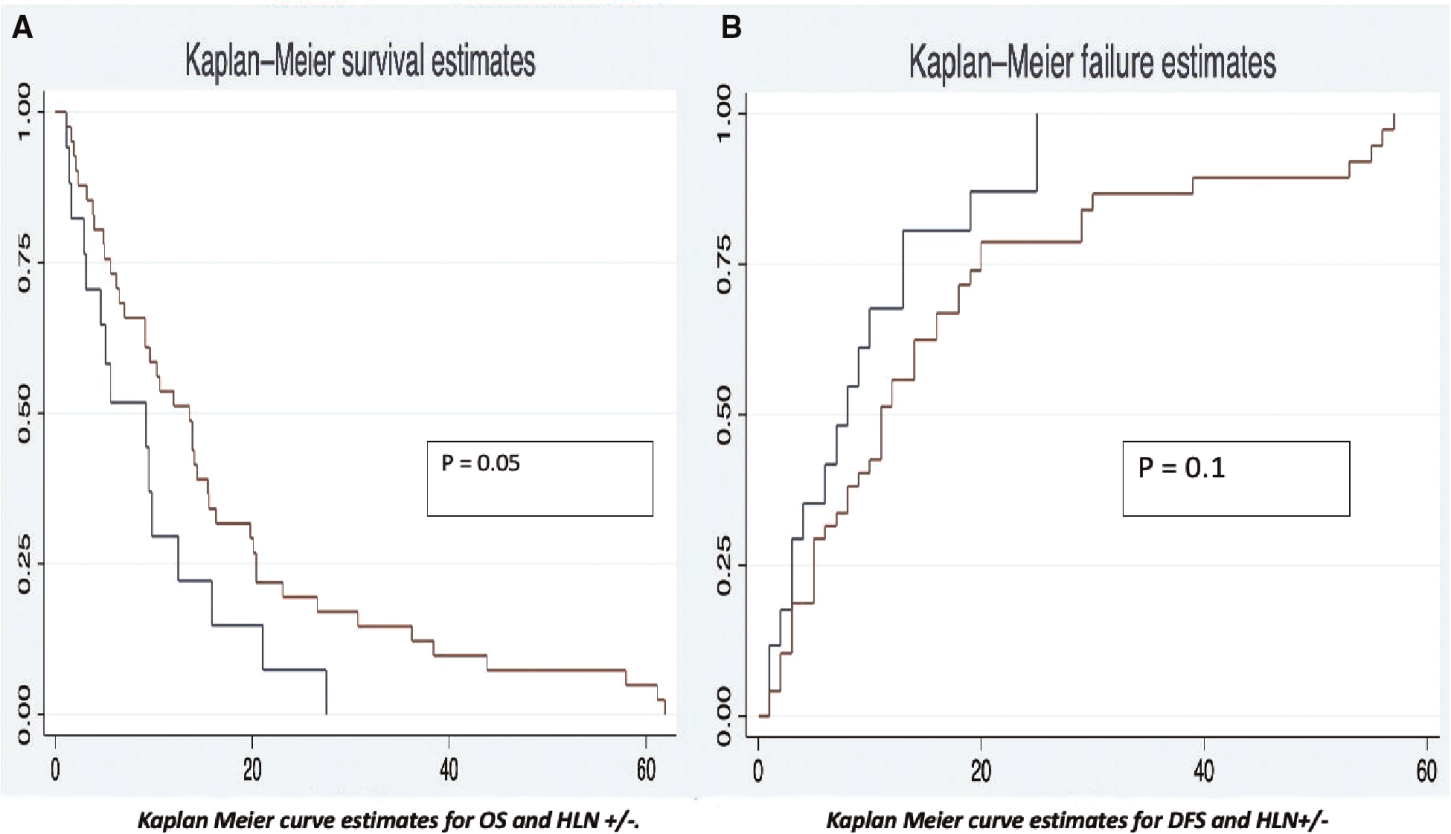
Kaplan meier curve estimates for OS and DFS (HLN +/−).

**Figure 3:**
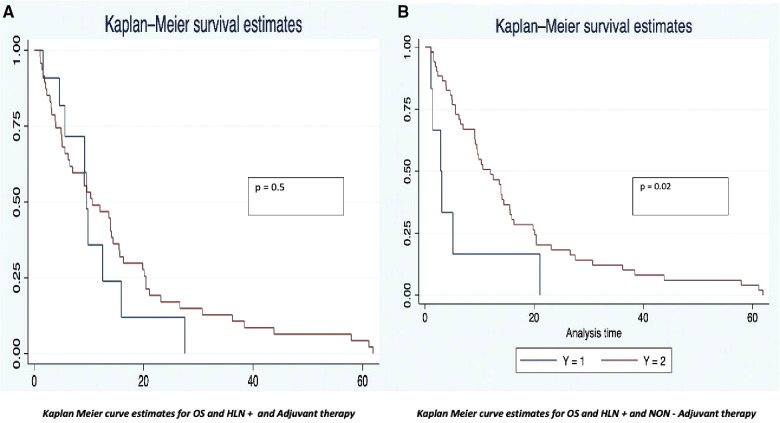
Kaplan meier curve estimates for OS (HLN +/− vs. Chemo/Non chemotherapy groups).

The OS was analyzed taking into consideration the HALN(8a) positive rate and its relation to the most relevant prognostic factors (vascular, perineural invasion, and margin of resection). For HALN(8a) positive cases (21,62% *n* = 24/111) the OS time was 12.5 months, vs. 20.4 in the negative node group (CI 95% 9–26). Figure 2 OS was significantly different in the adjuvant chemotherapy group analysis: for patients that did not receive adjuvant chemotherapy with positive HALN(8a) (62,5% *n* = 15/24), the median OS time was 12,5 months compared with 20,5 months in those who did receive adjuvant chemotherapy (36,5% *n* = 9/24). (Cox regression *p* = 0.02, HR 0.3, CI 95%) Kaplan Meier curves are displayed in Figure 3.

Overall survival was analyzed depending on the pathology report. In this analysis, pancreatic ductal adenocarcinoma with HALN(8a) compromise shows a statistical relationship with overall survival (*p* 0.00 CI 95% 0.0–0.99 HR 9.1). Ampulla of Vater tumors were excluded from this analysis due to the small sample size.

Regarding other prognostic variables, the LogRank test showed, for positive vascular invasion an OS time of 15.5 months, compared to 27.5 months (CI 95% 12–36) in the negative group. For perineural invasion, the positive group had an OS time of 15.9 months vs. the negative one of 30,7 months (CI 95% 15–61).

In the Cox regression model including relevant variables [HALN(8a) +/−, Vascular invasion +/−, perineural invasion +/−, resection margins +/−, and our new cutoff value for LNRR] statistical association was present between the positive HLA(8a), the cutoff of lymph node ratio resection, and vascular invasion (CoxRegression *p* = 0.05 HR 0.5; 0.003 HR1.8; *p* = 0.02 HR 0.4 CI 95%, respectively). LogRank tests and cox regression results are displayed in Table 3.

– Lymph node subgroup analysis.

**Table 3:** Log rank and Cox regression analysis for OS.

Variable	Logrank 95% CI	Cox Proportional 95% CI	Chi Square (HR)
**Haln ±**	***p* 0.05**	***p* 0.05**	***3.1*** (0.5)*
Tumoral size > 2 cm	*p* 0.09	*p* 0.09	3.1 (1.8)
Perineural invasion	*p* 0.05	*p* 0.06	3.8 (0.5)
** Vascular invasion **	***p* 0.02**	***p* 0.02**	***5.2*** *(*0.4)*
Resection margins	0.92	0.92	0.01 (0.9)
** LNRR **	***p* 0.001**	***p* 0.003**	***9.67*** (***1.8)***
***Adjuvant therapy/HLA Node (±)***	***p* 0.05**	***p* 0.02**	***4.5*** *(*0.3)*

* Statistically significant value.

Evaluation of OS was performed according to each lymph node group. Significant differences were found for each group. For patients in peripancreatic (+) and HALN (+), the median overall survival time was 20 months (IQR 10;50), compared with peripancreatic (+) and HALN (−) with a median OS of 35 months (IQR 20;47). In patients with peripancreatic (−) and HALN (−), the median OS was 46 months (IQR 20;74).

**Figure 4:**
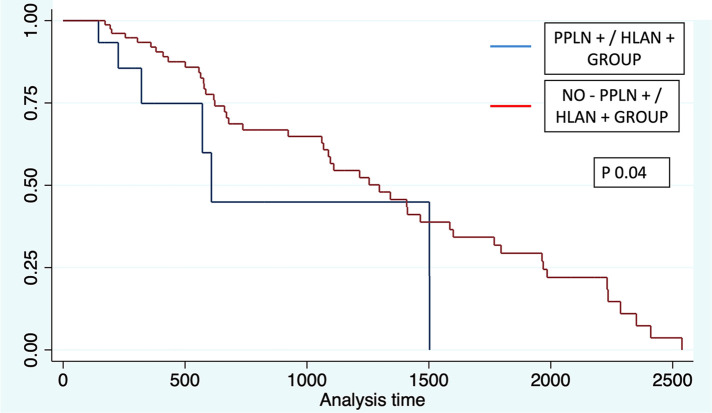
KAPLAN–MEIER curve estimates for OS and lymph node subgroup analysis.

Log-rank tests were performed for each group in order to evaluate the association with OS. Peripancreatic (+)/HALN (+) and Peripancreatic (−)/HALN (−) were related with significant statistical value with OS (Chi^2^ = 3.46; *p* 0.06 and Chi^2^ = 4.41; *p* 0.03 respectively). Further Cox-Proportional evidence that patients with peripancreatic (+)/HALN (+) have an increased 2.13-fold risk to have lesser OS with statistically significant value (HR 2.13 *p* 0.04 95% CI 0.94–4.84). (See Figure 4).

### Disease-Free survival time

The disease-free survival time also had differences between the analysis groups. The positive HALN(8a) group patients have a DFS time of 13 months, compared with 20 months (CI 95% 12–22) in the negative HALN(8a) group, with a value as an independent factor to DFS (*p* = 0.09), but failed to be significative in the Cox proportional regression model. DFS was significantly different in the adjuvant chemotherapy group analysis: HALN(8a) + and adjuvant chemotherapy + patients (36,5% *n* = 9/24) have had a prolonged DFS time: (29 months), compared with the non-chemotherapy (62,5% *n* = 15/24) group: (13 months). In the Cox proportional regression model, DFS in HALN(8a) negative patients and adjuvant chemotherapy + did not differ significantly compared to HLAN(8a) negative and non-chemotherapy group (18 months CI 95% 17–20 vs. 19 months CI 95% 6–21, respectively).

Pancreatic ductal adenocarcinoma with HALN(8a) compromise was not related to the DFS period according to cox proportional analysis (*p* 0.5 CI 95% 0.6–2.4 HR 1.1). However; ampulla of Vater carcinoma with HALN compromise was related with statistical significance with DFS period (*p* 0.04 CI 95% 1.07–56 HR 7,7).

Regarding other relevant prognostic factors, positive vascular invasion presented a DFS of 14 months vs. 53 months in the negative group (CI 95%, 14–60). In the positive perineural invasion group, the DFS was 18 months, and in the negative one, the DFS was 53 months (CI 95%, 15–55). Kaplan Meier estimates curves for OS and DFS are displayed in Figure 3.

Cox proportional regression showed as relevant variables the lymph node ratio cutoff (*p* = 0.008, HR = 1.5 CI 95%), tumoral size > 2 cm (*p* = 0.04 HR = 2.1 CI 95%), and vascular invasion (*p* = 0.02 HR = 0.4, CI 95%). In this analysis, HALN(8a) + does not show a significant relationship with DFS. Log-Rank tests and cox regression results are displayed in Table 4.

– Lymph node subgroup analysis.

**Table 4:** Log rank and Cox regression analysis for DFS.

Variable	Logrank 95% CI	Cox proportional 95% CI	Chi square (HR)
Haln +	*p* 0.09	*p* 0.1	—
** Tumoral size > 2 CM **	***p* 0.03**	***p* 0.04**	***4.63*** *(*2.1)*
Perineural invasion	*p* 0.1	*p* 0.1	—
** Vascular invasion **	***p* 0.001**	***p* 0.02**	***5.71*** *(*0.4)*
Resection margins	0.6	—	—
** LNRR **	***p* 0.01**	***p* 0.008**	***6.01*** *(*1.5)*
Adjuvant therapy/HLA Node (+)	*p* 0.2	*p* 1.0	

* Statistically significant value.

Evaluation of DFS was performed according to each lymph node group. For patients in peripancreatic (+) and HALN (+), the median disease-free survival time was 6 months (IQR 3;10), compared with peripancreatic (+) and HALN (−) with a median DFS of 8 months (IQR 5;17). In patients with peripancreatic (−) and HALN (−), the median DFS was 14 months (IQR 6;25).

**Figure 5:**
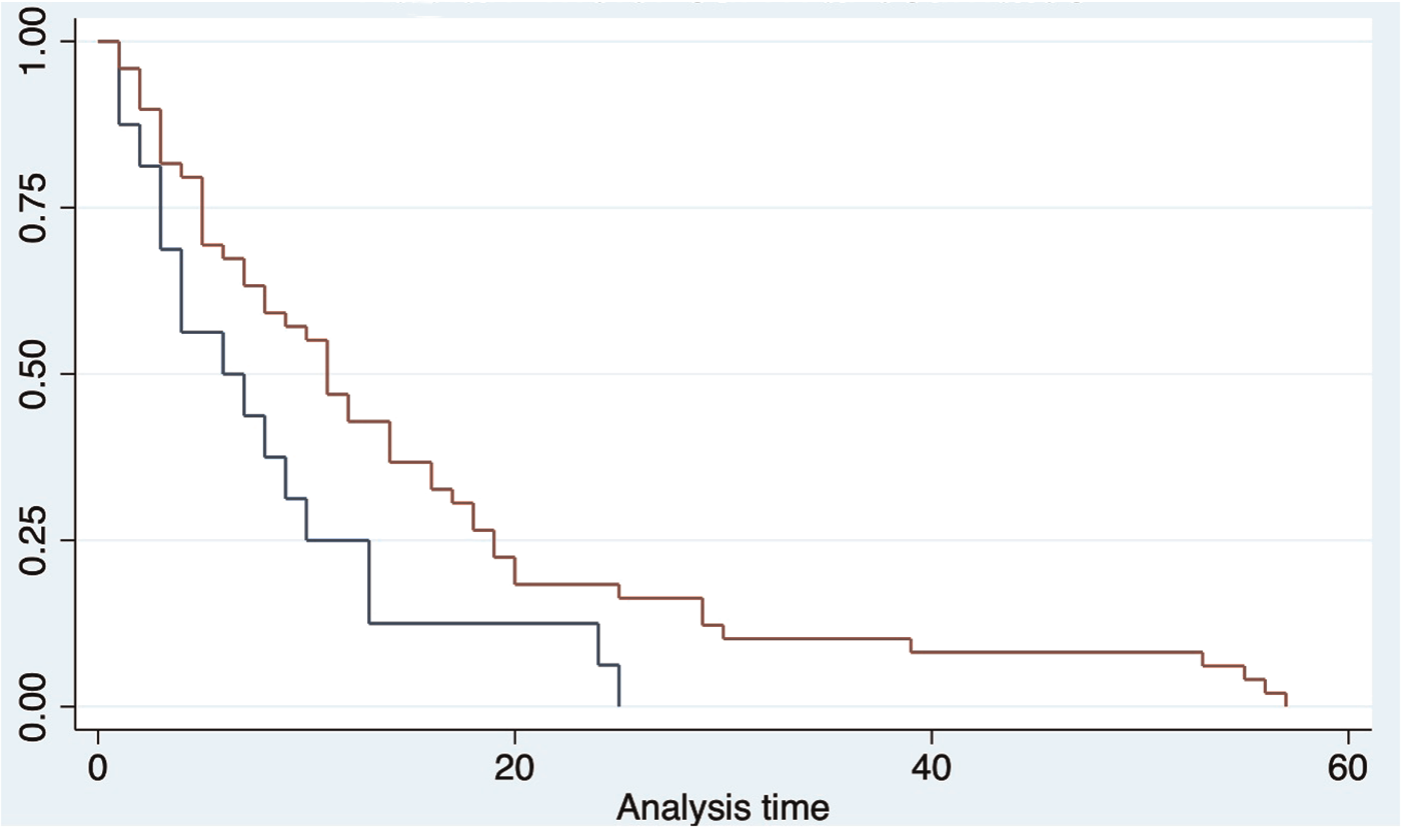
Kaplan–meier survival estimates.

LogRank tests were performed for each group in order to evaluate the association with DFS. Peripancreatic (+)/HALN (+) and Peripancreatic (−)/HALN (−) were related with significant statistical value with DFS (Chi^2^ = 4.81; *p* 0.02 and Chi^2^ = 5.77; *p* 0.01 respectively). Further Cox-Proportional evidence that patients with peripancreatic (+)/HALN (+) have an increased 1.82-fold risk to have lesser DFS with statistically significant value (HR 1.86; *p* 0.03 95% CI 1.04–3.34) (See Figure 5).

## Discussion

Given the ominous prognosis in periampullary cancers, many prognostic factors are identified as relevant in the survival outcomes (11–24). Adequate Lymph node dissection is one of the most relevant prognostic factors, however, these cancers have multiple routes for lymphatic dissemination and periampullary tumors lack a single draining or sentinel lymph node that can be biopsied preoperatively to detect early metastasis (25). In fact, a study in 2007 performed on 28 patients discarded the use of the 8a node as a sentinel node (26). However, Recently, HALN(8a) study has gained interest and multiple retrospective studies describe the relevance as a prognosis factor (12–14). MicroRNAs panel assay in HALN(8a) biopsies found shorter recurrence-free survival when positive for MicroRNAs-10b in periampullary carcinomas (25).

This study found a reduction of OS in patients with positive HALN(8a). OS was 20.4 months in negative HALN(8a) compromise vs. 12.5 months in the positive group, nonetheless with no statistical difference (*p* = 0.05). These results are similar to those reported by Cordera et al. who described in 175 pancreatic cancer patients a median overall survival time of 14.7 months in a positive HALN(8a) compared with 16.1 months in peripancreatic node involvement and 22.9 months in all negative nodes (15). In addition, some authors described in a retrospective cohort of 147 patients with PC, a difference in OS in a group of patients divided according to the peripancreatic lymph node compromise in relation to HALN compromise [OS: 26 months PPLN−/HALN−; 17 months in the PPLN+/HALN−, and 13 months in the setting of HALN + (*p* = 0.017)]. HALN(8a) + has a 2.94-fold reduction in OS and 2.66-fold reduction in DFS (14). These results are similar to those observed in our population. (In HLA node-positive a 2.0-fold OS reduction and 1.77-fold in DFS); Our subgroup analysis demonstrates significant differences in overall survival comparing PPLN (+)/HALN (+) vs. PPLN (+)/HALN (−) (20 vs. 25 months) (*p* 0.04), thus reflecting the negative influence of HLAN positivity in patients who underwent Whipple procedure for malignant conditions.

Among relevant prognosis factors in OS included in the primary analysis (HALN positivity, tumoral size, perineural and vascular invasion, resection margins, LNRR, and HALN compromise with adjuvant therapy), the log-rank test showed statistical relationship as independent factors of vascular invasion, LNRR and the positivity of HALN Node in patients that receive adjuvant therapy; nonetheless, in Cox proportional analysis, HALN node positivity alone, failed to reach statistical significance in relationship with OS, however, vascular invasion, LNRR, and HALN in patients with adjuvant therapy show statistical relationship with overall survival increasing the risk of mortality in 5.2, 9.67, and 4.5 times respectively.

In the primary evaluation of DFS, significant variables were LNRR + (*p* = 0.01, chi^2^ 6.01), HALN + (*p* = 0.09, chi^2^ 3.32), tumoral size > 2 cm (*p* = 0.03 chi^2^ 4.6), vascular invasion (*p* = 0.002 chi^2^ 5.71), perineural invasion (*p* = 0.1 chi^2^ 3.36), and resection margins (*p*-value 0.6 chi2 0.01). The multivariate analysis found a strong association of the lymph node ratio cutoff with a significant statistical value (*p* = 0.008, CI 95%), tumoral size > 2 cm (*p* = 0.02 CI 95%), and vascular invasion (*p* = 0.02 CI 95%). HALN(8a) compromise failed to confirm relevance in DFS Cox proportional hazard model. Data reported by Phillips et al. show that patients with lymphatic involvement (peripancreatic) had an OS of 19,5 months. However, patients without lymph node involvement had a median of 40,2 months (*p* < 0,001) (5) They didn't find a difference between OS and DFS of patients with positive HALN(8a) (mean 18,4 months and 10,6 months) and negative HALN(8a) patients (mean 19,7 months and 11,6 months) (5). One of the limitations of that study is the lack of evidence in pathology reports of HALN, and this could impact the final results.

Taking into consideration that patients with positive HALN(8a) have a significantly greater tumoral load and worse tumoral biology, some authors have compared the prognosis with peritoneal carcinomatosis (11, 12). Connor et al., found that the OS for patients with positive HALN(8a) was poor when compared to those with negative HALN(8a) (mean of 197 vs. 470 days, *p* = 0,003), which is why it could be compared with patients with unresectable metastatic disease (mean of 98 days, *p* = 0,072), thus considering the compromise of HALN(8a) an important predictor of occult metastatic disease. Nonetheless, current data does not support the involvement of HALN(8a) as a non-resectability criterion (12, 24).

Tumoral biomarkers' relevance in diagnosis, prognosis, and surgical decision-making processes is still a matter of debate (29, 30). Ca 19-9 it's one of the most studied; Santucci et al. (31, 32), found that major levels of CA 19-9 in patients with locally advanced pancreatic cancer or metastatic disease compared with those with resectable disease; however, limitations among the use of CA 19-9 in clinical practice includes that almost 50% of patients with tumors < 2 cm do not elevate any tumoral biomarker and for that reason, currently there is still a lack of evidence in terms of prognosis (30). Our study did not find any statistical difference between CA 19-9 levels in patients with positive HLA compared with the negative group.

As is known, multimodal treatment including surgery and chemotherapy is a cornerstone in the management of pancreatic cancer, Liu et al. (30) described the well-known impact on the prognosis of patients that receive chemotherapy after an R0 surgery, with an OS of 20 months compared with the non-chemotherapy group with 11.6 months. A large proportion of patients from this study did not receive adjuvant chemotherapy 42.34% (*n* = 47) because of health insurance issues or personal decisions that might cause a negative impact on the results. Therefore, a differential analysis of patients was performed in separate groups of patients who receive or do not adjuvant chemotherapy. Patients with HALN(8a) compromised plus chemotherapy have an OS of 20.5 months vs. non-chemotherapy with 12.5 months (*p* = 0.02) (26, 33, 34). The relevance of HALN(8a) is therefore highly influenced by other prognostic factors, like chemotherapy which is a cornerstone in periampullary cancer treatment.

There is no doubt about the importance of lymph node dissection, until now, a dissection of between 10 and 16 nodes is recommended in PD. A large retrospective cohort from the Surveillance, Epidemiology, and End Results (SEER) database, described an improved median overall survival from patients with more than 16 nodes harvested in comparison to the subgroup with less than 10 nodes harvested (117 months vs. 40 months, *p* < 0.001) (34–36). However, the dissection of the HALN(8a) is under debate as a preoperative or intraoperative tumoral progress. pancreatic cancer seems to be most affected by this node positivity. Few studies discuss the impact of lymph node retrieval in non-pancreatic periampullary cancers. However, given the histologic uncertainty in some cases, and the increasing evidence regarding the relevance of this node, we recommend sending it to pathology in all cases.

Limitations of our study include the retrospective nature of the study and the small sample size of patients receiving chemotherapy. None of our patients received neoadjuvant therapy and this can also influence the results. However, subgroup analysis was performed to decrease bias. To the best of our knowledge, this is the first report in Colombia evaluating survival analysis in the context of pancreatic cancer, reports in Latin American countries are limited to some reviews (27, 28).

## Conclusion

This retrospective study with prospective data from an HPB center shows data and statistical analysis suggesting that HALN(8a) compromise in properly treated patients should be considered as an impact factor in survival. We cannot recommend stopping surgery in patients with positive HALN(8a). However, it could suggest the aggressive biology of the tumor based on the reduced OS. HALN(8a) metastases and an LNRR bigger than 0.6 are relevant prognostic factors in periampullary cancer to predict reduced OS and DFS respectively. Notwithstanding the worldwide literature, our study does not find any relationship between the preoperative value of CA 19-9 and HALN positivity. Further prospective studies are needed to confirm these results.

## Data Availability

The raw data supporting the conclusions of this article will be made available by the authors, without undue reservation.
